# Characterization of *Alternaria porri* causing onion purple blotch and its antifungal compound magnolol identified from *Caryodaphnopsis baviensis*

**DOI:** 10.1371/journal.pone.0262836

**Published:** 2022-01-20

**Authors:** Min Young Kim, Jae Woo Han, Quang Le Dang, Jin-Cheol Kim, Hun Kim, Gyung Ja Choi

**Affiliations:** 1 Center for Eco-friendly New Materials, Korea Research Institute of Chemical Technology, Daejeon, Korea; 2 Department of Agricultural and Biological Chemistry, Chonnam National University, Gwangju, Korea; 3 Research and Development Center of Bioactive Compounds, Vietnam Institute of Industrial Chemistry, Hanoi, Vietnam; 4 Department of Medicinal Chemistry and Pharmacology, University of Science and Technology, Daejeon, Korea; Tocklai Tea Research Institute, INDIA

## Abstract

*Alternaria porri* (Ellis) Clf. causes purple blotch disease on *Allium* plants which results in the reduction of crop yields and quality. In this study, to efficiently find natural antifungal compounds against *A*. *porri*, we optimized the culture condition for the spore production of *A*. *porri* and the disease development condition for an *in vivo* antifungal assay. From tested plant materials, the methanol extracts derived from ten plant species belonging to the families Cupressaceae, Fabaceae, Dipterocarpaceae, Apocynaceae, Lauraceae, and Melastomataceae were selected as potent antifungal agents against *A*. *porri*. In particular, the methanol extract of *Caryodaphnopsis baviensis* (Lec.) A.-Shaw completely inhibited the growth of *A*. *porri* at a concentration of 111 μg/ml. Based on chromatographic and spectroscopic analyses, a neolignan compound magnolol was identified as the antifungal compound of the *C*. *baviensis* methanol extract. Magnolol showed a significant inhibitory activity against the spore germination and mycelial growth of *A*. *porri* with IC_50_ values of 4.5 and 5.4 μg/ml, respectively. Furthermore, when magnolol was sprayed onto onion plants at a concentration of 500 μg/ml, it showed more than an 80% disease control efficacy for the purple blotch diseases. In terms of the antifungal mechanism of magnolol, we explored the *in vitro* inhibitory activity on individual oxidative phosphorylation complexes I–V, and the results showed that magnolol acts as multiple inhibitors of complexes I–V. Taken together, our results provide new insight into the potential of magnolol as an active ingredient with antifungal inhibitory action to control purple blotch on onions.

## Introduction

Onions (*Allium cepa* L.) are one of the major vegetable crops grown in widespread areas and consumed throughout the world [1]. *Alternaria porri* (Ellis) Cif. is a causal agent of purple blotch disease on *Allium* plants such as onion, leek, and garlic plants [2] and causes severe damage to onions, resulting in losses in seed and bulb production [3]. The symptoms on onion leaves by this fungal species appear as sunken purple lesions that are often elliptical with a yellow to pale‐brown border [4]. Additionally, the Alternaria toxins such as tentoxin, silvaticol, and porritoxinol found in plants infected by *A*. *porri* could pose a potential health risk to human [5].

Synthetic chemical fungicides (e.g., mancozeb) have been widely used to control *Alternaria* diseases on various crops and vegetables [6]. The chemical controls using synthetic fungicides have been considered as one of the most efficient and consistent means to improve both yield and quality of crops. Nevertheless, the continuous use of synthetic chemicals is toxic to non-target organisms and causes environmental problems [7]. Moreover, the emergence of fungicide-resistant pathogens renders fungicides ineffective, and the increased awareness of food safety has been provoking strict regulatory policies on their use [8, 9]. The growing demands for sustainable agriculture and public concern for human health and the environment have led to finding alternatives to conventional synthetic fungicides. Biological controls using natural products (e.g., plant extracts and antagonistic microorganisms) have been considered to overcome the shortcomings of conventional chemical controls [10, 11]. Notably, various secondary metabolites of plants and microbes exhibit antifungal activity with unique chemical structures and modes of action distinct from synthetic fungicides [12]. Some natural compounds have also been used as lead molecules to develop synthetic fungicides or directly used as biochemical fungicides [13].

In terms of natural antifungal resources against *Alternaria* spp., it has been reported that the essential oils of *Thymus vulgaris*, *Cinnamomum zeylanicum*, and *Eugenia caryophyllus* are active against *Alternaria alternata*, a causal agent of *Alternaria* brown spot [14, 15]. Garlic extract is also effective in the suppression of seed-borne infection of *Alternaria* spp. in carrots [16]. In addition to plant extracts and essential oils, several phytochemicals such as morinols, *β*-sitosterol, and *β*-sitosteryl linoleate have been identified as an active compound against *Alternaria* spp. [17, 18]. Microbes have been also reported to be effective against *Alternaria* spp. For example, the post-harvest decay of cherry tomatoes caused by *A*. *alternata* was significantly reduced by the treatment of rhamnolipid produced by *Pseudomonas aeruginosa* [19]. The mycoparasitic fungus *Trichoderma harzianum* Th-3 and cyanobacterial strains of *Nostoc* and *Oscillatoria* were also effective in reducing the plant disease severity of onion purple blotch caused by *A*. *porri* [20, 21].

For *in vitro* and *in vivo* antifungal assays, enough spores from the target pathogens are required as an inoculum. Considering that *A*. *porri* produces relatively few spores on agar media, physical treatments such as UV irradiation and mycelial wounding have been considered as an efficient method for increasing spore production [22]. Therefore, one of the goals of this study was to establish the optimized conditions for the spore production of *A*. *porri* and the development of onion purple blotch to help discover antifungal substances from natural resources. Based on this system, we investigated the antifungal activity of various Vietnamese plant materials, and the methanol extract of *Caryodaphnopsis baviensis* (Lec.) A.-Shaw was found to have an antifungal activity against *A*. *porri*. This study also aimed 1) to isolate and identify the active compounds from the *C*. *baviensis* extract, 2) to evaluate the efficacy of the identified active compounds against *A*. *porri* and other plant pathogens, and 3) to understand the modes of inhibitory actions of the compounds.

## Materials and methods

### Fungal strain, culture medium, and sporulation

Fungal pathogens used in this study were obtained from the Korean Agricultural Culture Collection (Suwon, Korea): four *A*. *porri* strains (KACC 40568, 42998, 43771, and 43001) and six fungal species (*Alternaria brassicicola* KACC 40036, *Botrytis cinerea* KACC 48736, *Colletotrichum coccodes* KACC 48737, *Fusarium oxysporum* f. sp. *lycopersici* KACC 40043, *Magnaporthe oryzae* KACC 46552, and *Phytophthora infestans* KACC 48738). These fungal species were maintained on a potato dextrose agar (PDA; BD Difco, San Jose, CA) plate at 25°C, except for *B*. *cinerea* and *P*. *infestans* incubated at 20°C [11]. For the spore production of *A*. *porri*, we used four different types of agar media: V8 juice agar (V8), carboxymethyl cellulose agar (CMCA), oatmeal agar (OA), and malt extract agar (MEA) media [11].

To investigate the sporulation of the four *A*. *porri* strains, each mycelial disk (8.5-mm diameter) of *A*. *porri* grown on PDA was inoculated on a V8 plate. After incubation for 7 days at 25°C, culture plates were kept in a moisture chamber (25°C, 80% relative humidity (RH), and 12-h photoperiod) for 2 days. Spores from the culture plates were harvested by adding sterile water and repeatedly scraping with a sterile brush, and then the spore suspension was passed through four layers of cheesecloth. To evaluate spore production depending on the environmental condition, we used different culture media (V8, PDA, OA, CMCA, and MEA) and light conditions (darkness and 12-h photoperiod). For physical stress on the culture plates, the surface of 7-day-old culture plates was scraped by a blade or brush and scratched into grid patterns with 2- and 5-mm spacing by a scalpel blade. All tests were performed two times with three replicates.

### Developmental condition of onion purple blotch

Onion plant (*Allium cepa* L.) cultivars Redoneball 1115 and Speedup were grown in a 72-hole plug tray containing a commercial nursery soil (Punong, Gyeongju, Korea) which was maintained in a greenhouse at 25 ± 5°C. As an initial method for purple blotch, the 4-leaf stage of onion plants were spray-inoculated with spore suspension (1 × 10^5^ spores/ml in a 0.025% Tween 20 solution (w/v)) of *A*. *porri*, and then, the inoculated plants were transferred into a humidified growth chamber (20°C). After 24 h of incubation, the inoculated plants were moved to a growth chamber (20°C, 80% RH, and 12-h photoperiod) and kept for a further 2- or 4-day incubation. To optimize the condition for disease development, the *in vivo* assay described above was modified as follows: by the seedling stages (2-, 3-, and 4-leaf stages), incubation temperature (17, 20, 25, and 30°C), inoculum concentration (0.04, 0.2, 1, and 5 × 10^5^ spores/ml), and incubation time in the humidified chamber (24 and 48 h). The disease severity based on the lesion area on the leaves was evaluated 3 or 5 days post inoculation (dpi). The efficacy of disease control was calculated with the following equation: disease control efficacy (%) = 100 × [1 –B / A], where A is the mean lesion area (%) on the leaves of the control plants, and B is the mean lesion area (%) on the leaves of the treated plants [12, 23]. All tests were performed two times with three replicates.

### Antifungal activity assay of the plant extracts

To explore the *in vitro* antifungal activity of the plant materials, we used the methanol extracts derived from Vietnamese medicinal plants kindly provided by Dr. Quang De Lang at the Vietnam Institute of Industrial Chemistry (VIIC, Hanoi, Vietnam). The plant species were identified by Dr. Tran Bach from the Institute of Ecology and Biological Resources (Hanoi, Vietnam), and voucher specimens were deposited in the laboratory of the VIIC. To prepare the methanol extracts, the collected plant materials were air-dried and finely powdered using a blender (Waring model 34BL97, New Hartford, CT). Each powdered plant material (100 g) was extracted twice with 1 L of methanol for 48 h at room temperature, and the extracts were filtered through Whatman No. 1 filter paper (Merck, Kenilworth, NJ, USA). The filtrates were concentrated to dryness using a rotary evaporator (100 r/min, 40°C) and then stored at 20°C until further study. For the *in vitro* antifungal activity assay, each plant extract was dissolved in dimethyl sulfoxide (DMSO) at a concentration of 100 mg/ml and initially screened with a concentration of 1000 μg/ml in potato dextrose broth (PDB; BD Difco) containing a spore suspension (1 × 10^5^ spores/ml) of *A*. *porri*. After 3 days of incubation at 25°C, the antifungal activity was determined by visual inspection of complete growth inhibition. The chemical fungicide mancozeb and 1% DMSO were used as a positive and negative control, respectively. The antifungal activity of the resulting plant extracts was further investigated based on the broth microdilution method using three-fold serial dilutions starting with 1000 μg/ml as described previously [24]. All assays were conducted twice with three replicates for each treatment.

### Chromatographic analysis

To isolate the active compound against *A*. *porri*, the methanol extract (17.2 g) of *C*. *baviensis* was suspended in 200 ml of distilled water and then sequentially partitioned with *n-*hexane, ethyl acetate, and *n*-butanol. The resulting three organic solvent layers and aqueous layer were concentrated *in vacuo* to afford the fractions of *n-*hexane (2.1 g), ethyl acetate (1.3 g), *n*-butanol (3.6 g), and water (4.6 g). The *n-*hexane fraction was applied onto a silica gel column with a stepwise gradient elution of chloroform/methanol (100:0, 98:2, 95:5, 90:10, 60:40, and 0:100, v/v), yielding six fractions H1–H6. Based on bioassay-guided fractionation, the active fraction H4 (1.6 g) was applied onto a C16/100 column (GE Healthcare, Chicago, IL) packed with a Sephadex LH-20 resin (Supelco, Bellefonte, PA) and then eluted with methanol at a flow rate of 0.2 ml/min, yielding eight fractions H41–H48. The antifungal fractions H46 (173.9 mg) and H5 (126.6 mg) were merged because they showed a similar band pattern on a TLC plate. The combined fraction was further purified by Sephadex LH-20 column chromatography, yielding a pure compound CB1 (***C****aryodaphnopsis* ***b****aviensis* compound **1**; 118.8 mg).

The ethyl acetate fraction was also applied onto silica gel column chromatography and eluted with a stepwise gradient of chloroform/methanol (100:0, 95:5, 90:10, 80:20, 70:30, and 0:100, v/v), yielding six fractions E1–E6. The active fractions E1 (467.9 mg) and E2 (176.7 mg) were merged and then applied onto a silica gel column with an isocratic elution of dichloromethane/methanol (95:5, v/v), yielding four fractions E11–E14. The active fraction E13 (206.4 mg) was finally purified using Sephadex LH-20 column chromatography, yielding a pure compound CB2 (154.1 mg).

### Spectroscopic analysis

The chemical structure of antifungal compounds CB1 and CB2 was determined by spectroscopic analyses and comparison with data in the literatures. The EIMS analysis was performed using a Shimadzu QP2020 GC/MS system (Kyoto, Japan), and the results were recorded on a single-quadruple mass spectrometer (Acquity QDa; Waters, Manchester, UK). The 1D and 2D nuclear magnetic resonance (NMR) spectra were recorded by a Bruker Advance 500 MHz spectrometer (Bruker BioSpin, Rheinstetten, Germany) in CD_3_OD (99.8 atom% D; Cambridge Isotope Laboratories, Tewksbury, MA, USA). Chemical shifts were referenced to the solvent peaks (δ_H_ 3.31 ppm and δ_C_ 49.0 ppm).

### Inhibitory assay for mycelial growth and spore germination

To investigate the effect of active compound CB1 on the mycelial growth of *A*. *porri*, a mycelial disc (5 mm in diameter) of *A*. *porri* was inoculated onto PDA medium containing 1.2, 3.7, 11, 33, and 100 μg/ml of CB1, and then, the radial growth of *A*. *porri* was measured at 7 days after inoculation. The PDA plates containing 1% DMSO and mancozeb were prepared as a negative and positive control, respectively. For the inhibitory effect of CB1 on the spore germination of *A*. *porri*, CB1 was serially diluted three-fold, starting with a concentration of 100 μg/ml in microplate wells containing a spore suspension (5 × 10^3^ spores/ml of PDB). After a 10-h incubation at 25°C, the number of germinated spores was counted by microscopic observation in a total of 100 spores. All experiments were conducted twice with three replicates. The experimental results were expressed as the half-maximal inhibitory concentration (IC_50_) values calculated by the online IC_50_ calculator tool of AAT Bioquest (https://www.aatbio.com/tools/ic50-calculator).

To determine the minimum inhibitory concentrations (MICs) of CB1, we used the broth microdilution method previously described [24]. Breifly, CB1 was dissolved in DMSO at a concentration of 20 mg/ml and serially diluted two-fold, starting with a concentration of 100 μg/ml in microplate wells. Spore suspension of plant pathogenic fungi was dispensed into each well so that the final concentration was 1 × 10^5^ spores/ml in PDB. The cycloheximide (Sigma-Aldrich) and 1% DMSO were used as a positive and negative control, respectively. After incubation at 25°C for 3 days, MICs were determined by visual examination and corresponded to the lowest concentration that caused complete growth inhibition. The MIC tests were conducted twice with three replicates for each treatment.

### Disease control efficacy assay

To explore the effects of CB1 on the control efficacy for onion purple blotch, CB1 (125, 250, and 500 μg/ml) was prepared by dissolving it in a 5% aqueous methanol solution, including 0.025% Tween 20. Each sample was sprayed onto the 4-leaf stage of onion plants (cultivar Redoneball 1115), and the treated plants were dried for 24 h at room temperature. The treated plants were inoculated with a spore suspension (1 × 10^5^ spores/ml in a 0.025% Tween 20 solution) of *A*. *porri* and then incubated for 24 h in a humidified growth chamber (20°C). The inoculated plants were re-transferred into a growth chamber (25°C, 80% RH, and 12-h photoperiod) for a further incubation of 4 days. The disease severity based on the lesion area on the leaves was evaluated at 5 dpi, and the efficacy of the disease control was calculated as previously described in this study. As positive and negative controls, we used the chemical fungicide mancozeb (50 and 100 μg/ml) and 5% aqueous methanol that included in a 0.025% Tween 20 solution. All experiments were conducted twice with three replicates for each treatment.

### Mitochondrial respiratory inhibitory activity assay

The inhibitory action of CB1 on mitochondrial respiration was investigated with the following method described by Han et al. (2020) [25]. Briefly, the growth inhibition of *Saccharomyces cerevisiae* A-139 [25] by CB1 was compared according to two different media: YG (1% yeast extract and 2% glucose) and NFYG (1% yeast extract and 1% glycerol). One day after the CB1 treatment, the optical density (OD_600_) of each well was measured using a microplate reader (Bio-rad, Hercules, CA, USA), and the yeast growth inhibition (%) was calculated as follows: [1 - (OD_600_ of treatment/OD_600_ of control)] × 100. As a negative and positive control, distilled water and fungicide kresoxim-methyl were used for this assay, respectively.

To investigate the inhibitory activity against five electron transport chain complexes I–V, we used the MitoTox Complete OXPHOS Activity Assay kit (Abcam, Cambridge, MA, USA) following the manufacturer’s instructions. Briefly, CB1 was suspended in each assay solution at a concentration of 0, 1, 5, 20, and 100 μM, and the resulting samples were directly added to each oxidative phosphorylation (OXPHOS) complex. Specific inhibitors for each complex were used as a positive control: rotenone (complex I), thenoyltrifluoroacetone (TTFA, complex II), antimycin A (complex III), potassium cyanide (KCN, complex IV), and oligomycin (complex V). Microplate wells coated with a null capture antibody (complexes I, II, IV, and V) or wells without mitochondria (complex III) were used as a background control. After the addition of CB1 with the assay solution to the 96-well microplates, the absorption of each well was immediately measured using a microplate reader (Bio-rad) at wavelengths of 340 nm (complexes I and V), 600 nm (complex II), and 550 nm (complexes III and IV) with intervals of 60 s for 2 h (complex I), 60 s for 1 h (complexes II, IV, and V), and 20 s for 5 min (complex III). The activity of each complex was determined by the rate of change in absorbance after background subtraction. All experiments were conducted twice with three replicates for each treatment.

### Statistical analysis

All experiments were performed in triplicate with two runs and expressed as the mean ± standard deviation. All data except for yeast growth comparison were subjected to one-way analysis of variance (ANOVA) followed by Duncan’s new multiple range test. The Student’s t-test was subjected to compare yeast growth inhibition in two different media YG and NFYG containing magnolol. Significant differences (*p* < 0.05) were indicated with different letters or asterisks.

## Results

### Effects of the culture medium, physical stress, and light on spore production of *Alternaria porri*

**Fig 1:**
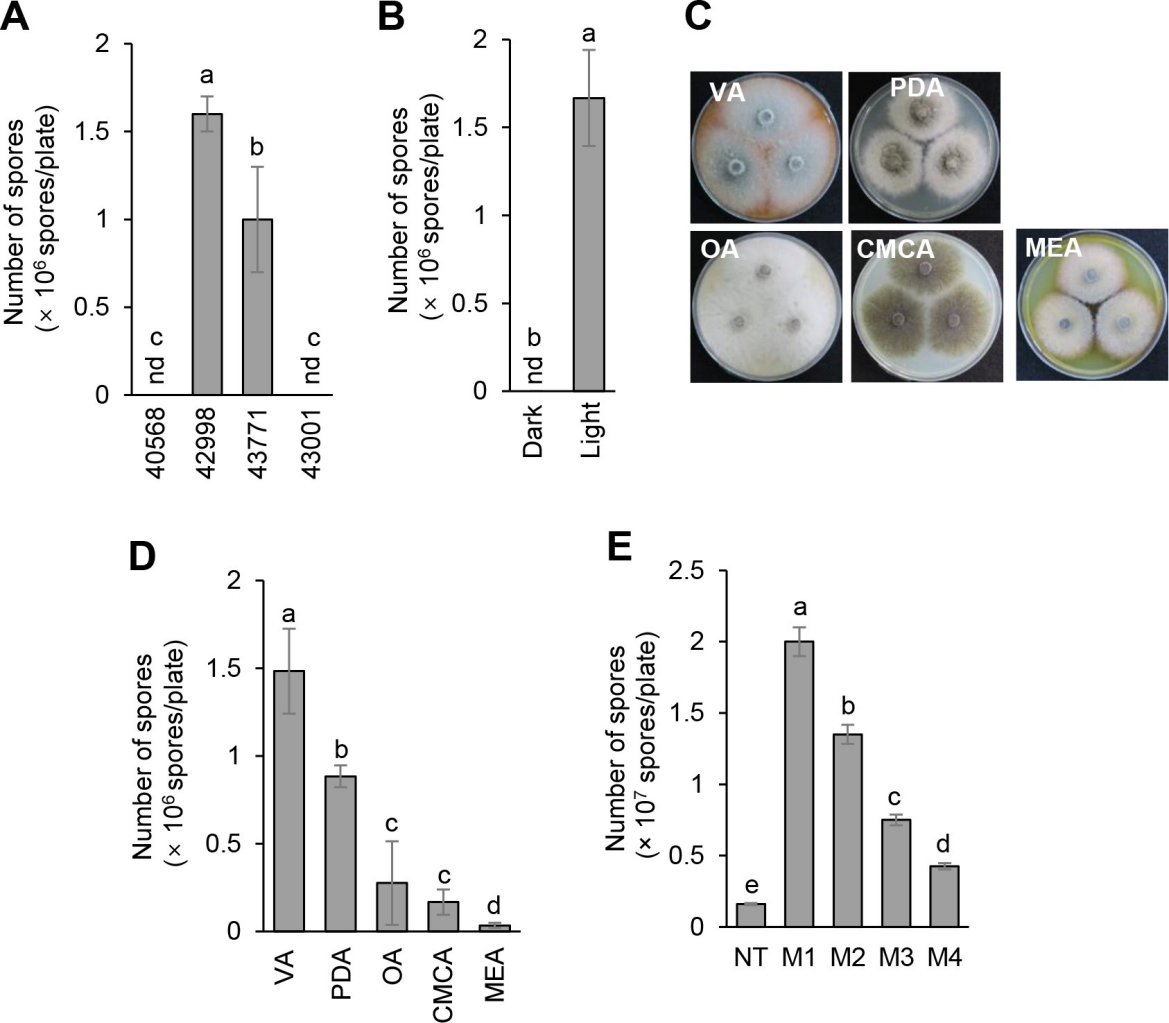
Spore production of *Alternaria porri*. (A) Comparison of spore production of four *A*. *porri* isolates grown on V8 medium for 9 days. (B) Effects of light on spore production of *A*. *porri* KACC 42998. (C and D) Spore production of *A*. *porri* KACC 42998 grown on different media for 9 days. V8, V8 juice agar medium; PDA, potato dextrose agar medium; OA, oatmeal agar medium; CMCA, carboxymethyl cellulose agar medium; MEA, malt extract agar. Photos of the culture plates were taken at 7 dpi. (E) Effects of physical stress on spore production. The culture surface of the V8 medium was not damaged mechanically (NT), scraped by a blade (M1) and brush (M2), or scratched into grid patterns with 2-mm (M3) and 5-mm (M4) spacing by a scalpel blade. The bars represent the mean ± standard deviation of two runs with three replicates. Different letters indicate significant differences at *p* < 0.05.

Spores of *A*. *porri* are not easily obtained under standard culture conditions, which affects the *in vitro* and *in vivo* assessment of antifungal resources [22]. To establish optimized conditions for the spore production of *A*. *porri*, the number of conidia harvested with sterilized distilled water from 9-day-old culture plates was measured by hemocytometer. When we investigated the spore amounts of four *A*. *porri* isolates grown on V8 medium for 9 days, a KACC 42998 strain produced the highest number of spores (1.6 × 10^6^ spores/plate) among the tested strains, whereas there were no spores observed from the cultures of the KACC 40568 and KACC 43001 strains (Fig 1A). After selecting the *A*. *porri* KACC 42998 strain, we investigated the effect of light stimulation on spore production. When 7-day-old culture plates grown in the dark were further incubated for 2 days in the dark or a 12-h photoperiod, the presence of light stimulation induced spore production, but not under darkness, suggesting that light stimulation is essential for the spore production of *A*. *porri* (Fig 1B). When the *A*. *porri* KACC 42998 strain was grown on different media such as V8, PDA, OA, CMCA, and MEA, we observed that spore production was the highest on V8 medium followed by the PDA, OA CMCA, and MEA medium (Fig 1C and 1D). To explore the effects of physical stress on spore production, the surfaces of the 7-day-old culture plates were scraped or scratched (S1 Fig) and then incubated for two more days with a 12-h photoperiod. The spore production of culture plates scraped by a blade was 10-fold more than that of the non-treated control (Fig 1E). However, the scratched culture plates produced less spores than that of the scraped culture plates (Fig 1E). Taken all together, our results show that the best spore production of *A*. *porri* was obtained with 7-day-old cultures grown on V8 medium scraped by a blade and further incubated under a 12-h photoperiod.

### Determination of the developmental condition for onion purple blotch disease

**Fig 2:**
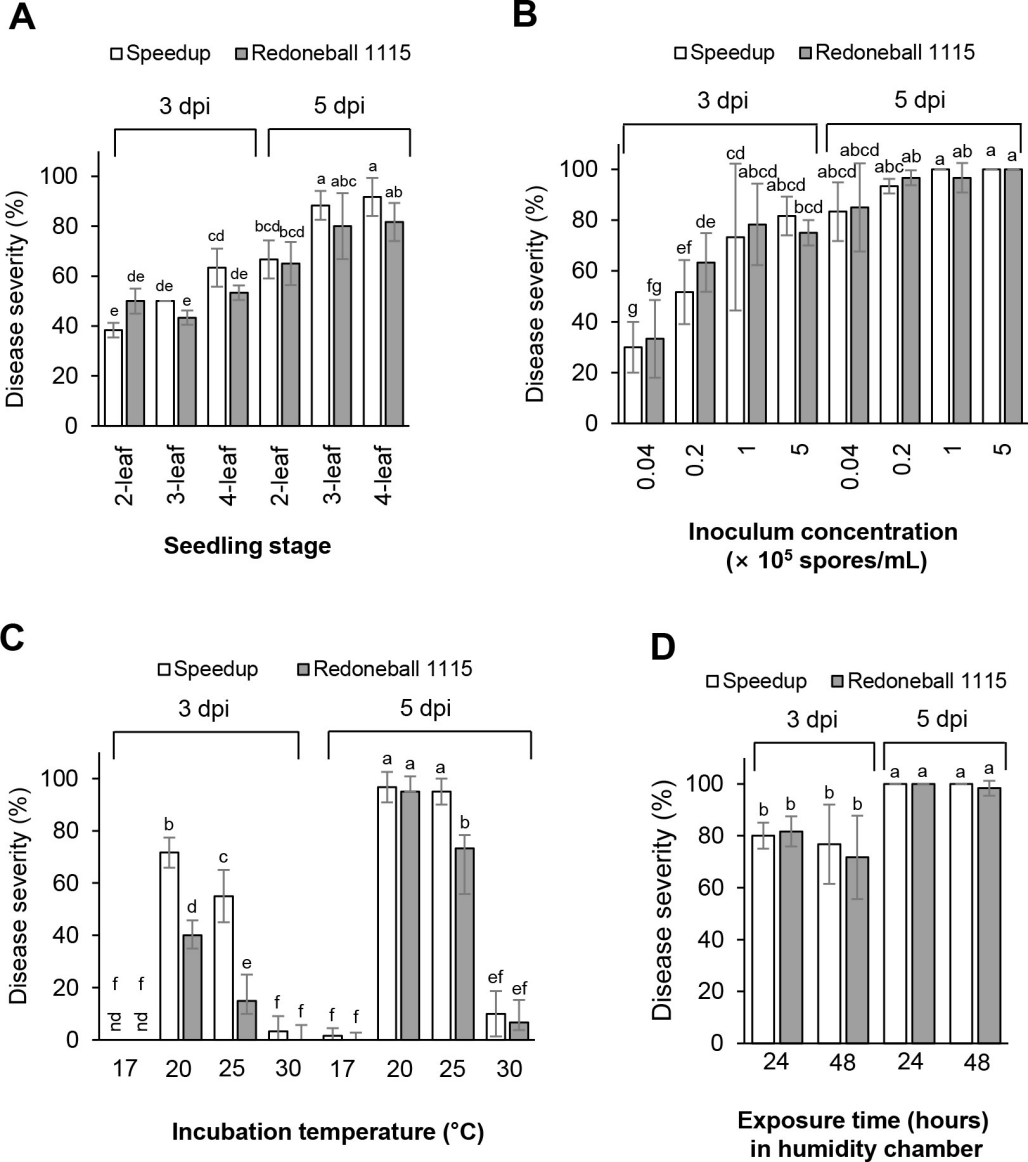
Disease development of onion purple blotch. Effects of leaf stage (A), inoculum concentration (B), temperature (C), and humidity (D) on the development of onion purple blotch. As host plants, two onion cultivars Speedup and Redoneball 1115 were used for the assay. The bars represent the mean ± standard deviation of two runs with three replicates. Different letters indicate significant differences at *p* < 0.05.

To determine the favorable condition for the development of onion purple blotch (S2 Fig), we inoculated a spore suspension of *A*. *porri* onto different leaf stages of the cultivars Speedup and Redoneball 1115. The disease severity of both younger seedlings was less than that of the older seedlings at both 3 and 5 dpi (Fig 2A): at 3 dpi, the 4-leaf stage of the onion plants Speedup and Redoneball 1115 was the most susceptible to *A*. *porri* with disease severity values of 63% and 53%, respectively (Fig 2A). Similarly, at 5 dpi, the 4-leaf stage of Speedup and Redoneball 1115 was the most susceptible to *A*. *porri* with disease severity values of 92% and 82%, respectively (Fig 2A). However, according to the leaf stages, there was no significant difference in disease severity between the two cultivars. For our further studies, we determined to use the 4-leaf stage of the onion plants.

For the inoculum concentration, the disease severity of the 4-leaf stage of both cultivars increased as the inoculum concentration was increased (Fig 2B). When a spore suspension of 5 × 10^5^ spores/ml was inoculated onto the plants, the disease severity of Speed-up and Redoneball 1115 at 3 dpi was two-fold higher than that of plants inoculated with a spore suspension of 0.04 × 10^5^ spores/ml. At 5 dpi, both cultivars exhibited severe purple blotch regardless of the inoculum concentration ranging from 0.04 × 10^5^ to 5 × 10^5^ spores/ml. However, considering the results of the disease severity at 3 dpi, a spore suspension with a concentration of 1 × 10^5^ spores/ml or higher seems to be suitable for the development of purple blotch.

To investigate the proper temperature for the development of purple blotch, we incubated the inoculated plants with a spore suspension of 1 × 10^5^ spores/ml at various temperatures (17, 20, 25, and 30°C). When the inoculated plants were incubated at 17 and 30°C, the purple blotch on both cultivars was poorly developed at 3 and 5 dpi (Fig 2C). However, when the inoculated onion plants were incubated at 20 and 25°C, both cultivars exhibited typical disease symptoms. At 20°C, the cultivar Speedup exhibited disease severity values of 71% and 96% at 3 and 5 dpi, respectively, and the cultivar Redoneball 1115 exhibited disease severity values of 40% and 95% at 3 and 5 dpi, respectively (Fig 2C). Therefore, when the inoculated plants were incubated at 20°C, the results showed that the cultivar Redoneball 1115 exhibited less disease severity than that of Speedup at 3 dpi although the disease severity of the Redoneball 1115 was similar to that of the Speedup at 5 dpi. However, the inoculated plants incubated at 25°C showed less disease severity than that of the inoculated plants incubated at 20°C, particularly in the cultivar Redoneball 1115 (Fig 2C). Additionally, when the effects of exposure time (24 vs. 48 h) in a humidified growth chamber on the development of purple blotch were investigated, there was no difference in the disease severity according to the exposure time of humidity: when exposed to humidity for 24 and 48 h, both cultivars exhibited a disease severity of 80% at 3 dpi (Fig 2D). Taken together, we determined that the infection and development of onion purple blotch were highly stimulated when the 4-leaf stage of onion plants inoculated with a spore suspension of 1 × 10^5^ spores/ml were exposed into a humidified chamber at 20°C for 24 h and then incubated under a 12-h photoperiod for 4 more days at 20°C with 80% RH.

### Selection of antifungal plant extracts and identification of the antifungal compound

**Fig 3:**
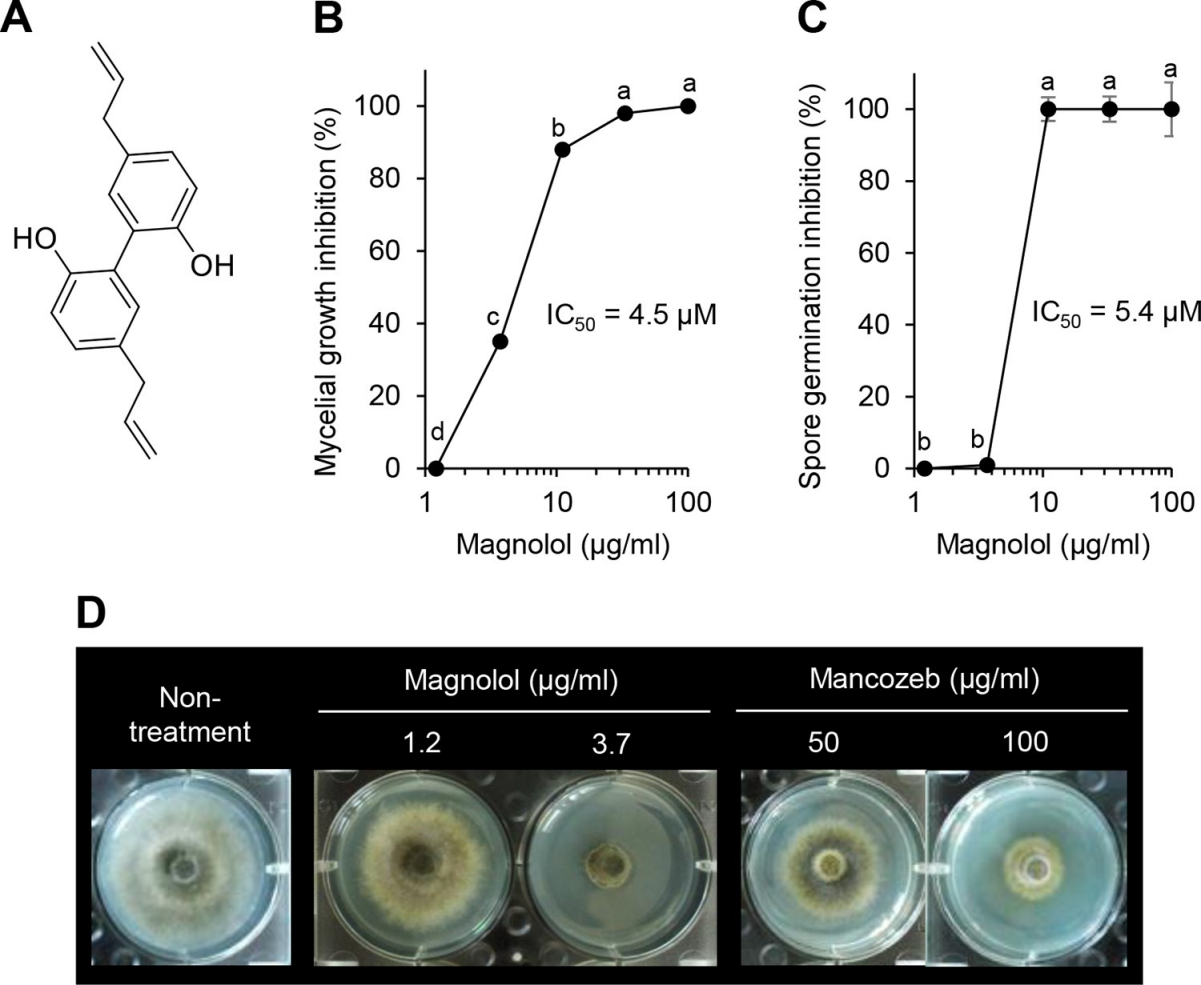
Effects of magnolol on spore germination and mycelial growth of *Alternaria porri*. (A) Chemical structure of the antifungal compound CB1 (magnolol). (B) Mycelial growth inhibition. The colony diameter was measured at 7 dpi, and the inhibition ratio (%) was calculated by comparison with non-treatment controls. (C) Spore germination inhibition. The number of germinated spores was counted in a total of 100 spores at 10 h after the magnolol treatment. (D) Representative colony morphology of *A*. *porri* grown on PDA supplemented with magnolol or mancozeb. For the non-treatment control, PDA containing 1% methanol was used. The values represent the mean ± standard deviation of two runs with three replicates. Different letters indicate significant differences at *p* < 0.05.

Of the tested plant extracts, ten plant species exhibiting an antifungal activity were initially selected based on an *in vitro* antifungal assay (Table 1). Among them, the methanol extract of *C*. *baviensis* leaves and stems was the most effective against *A*. *porri*, with the lowest MIC value of 111 μg/ml (Table 1). To isolate antifungal compounds from the methanol extract of *C*. *baviensis*, we performed chromatography analyses based on antifungal activity-guided fractionation (S3 Fig). Consequently, the antifungal compounds CB1 (118.8 mg) and CB2 (154.1 mg) were obtained as colorless needles from the *n-*hexane and ethyl acetate fractions derived from the *C*. *baviensis* methanol extract, respectively. The UV λmax values of CB1 were 220 and 289 nm. The EIMS and ESIMS spectra of CB1 showed molecular ions at m/z 266 M^+^ and m/z 265 [M—H]^-^, respectively. The NMR data of compound CB1 (S1 Table) completely agreed with those of magnolol (5,5’-di-2-propenyl-1,1’-biphenyl-2,2’-diol; C_18_H_18_O_2_), which is a neolignan that originates from the *Magnolia* spp. [26, 27]. Because of the symmetrically bridged biphenyl structure of magnolol (Fig 3A), the ^13^C NMR spectrum of CB1 exhibited only nine carbon signals that were assigned to three quaternary sp^2^ carbons at δ_C_ 153.2 (C-1), 127.6 (C-2), and 132.7 (C-4), four methine sp^2^ carbons at δ_C_ 139.4 (C-8), 133.2 (C-3), 129.8 (C-5), and 117.4 (C-6), one methylene sp^2^ carbon at δ_C_ 115.6 (C-9), and one methylene sp^3^ carbon at δ_C_ 40.4 (C-7). In the HMBC spectrum (S4 Fig), the correspondence of the ^1^H NMR signals was attributed to an aliphatic methylene proton at δ_H_ 3.32 (d, *J* = 6.9, 1.6, H-7), terminal methylene protons at δ_H_ 5.01 (ddt, *J* = 17.0, 2.2, 1.6, H-9a) and 5.05 (ddt, *J* = 2.4, 0.5 Hz, H-9b), an olefinic methine at δ_H_ 5.96 (ddt, *J* = 16.8, 10.1, 6.7, H-8), and three aromatic protons at δ_H_ 6.85 (d, *J* = 8.1), 7.02 (ddt, *J* = 8.1, 2.3, 0.6, H-5) and 7.03 (dt, *J* = 2.4, 0.5, H-3). Furthermore, we found that another active compound CB2 was identical to CB1 based on the spectroscopic analyses.

**Table 1:** Minimum inhibitory concentrations (MICs) of the methanol extracts from the leaf and stem extracts of Vietnamese plants against *Alternaria porri*.

Family	Scientific name	MIC (μg/ml)
Cupressaceae	*Cunninghamia lanceolata* (Lamb.) Hook	1000
Fabaceae	*Dalbergia spinosa* Roxb.	333
Dipterocarpaceae	*Hopea chinensis* (Merr.) Hand.-Mazz.	333
	*Hopea hainanensis* Merr. & Chun.	1000
Apocynaceae	*Heterostemma suberosum* Costantin	1000
Lauraceae	*Caryodaphnopsis baviensis* (Lec.) Airy Shaw	111
	*Litsea baviensis* Lecomte.	333
	*Litsea citrata* Blume	333
Melastomataceae	*Blastus eglandulosus* Stapf ex Spare	1000
	*Pycnarrhena poilanei* (Gagnep.) Forman	333

### *In vitro* and *in vivo* antifungal activity of magnolol against *Alternaria porri*

To examine the *in vitro* antifungal activity of the identified compound magnolol (CB1), we investigated the inhibitory effects on the mycelial growth and spore germination of *A*. *porri*. When PDA and PDB media were supplemented with magnolol, it had an inhibitory activity against both the mycelial growth and spore germination of *A*. *porri* with IC_50_ values of 4.5 and 5.4 μg/ml, respectively (Fig 3B and 3C). Moreover, the mycelial growth and spore germination of *A*. *porri* were completely inhibited with MIC values of 100 and 11 μg/ml, respectively. Notably, the inhibitory activity of magnolol on mycelial growth was more potent than that of the broad-spectrum fungicide mancozeb used as a positive control in this study (Fig 3D). In addition to *A*. *porri*, magnolol had a broad-spectrum antifungal activity against phytopathogenic fungi such as *B*. *cinerea*, *C*. *cocodes*, *F*. *oxysporum* f. sp. *lycopersici*, and *M*. *oryzae* with MIC values ranging from 3.1 to 25 μg/ml, which was comparable to the antifungal activity of cycloheximide used as a control (Table 2).

**Table 2:** Minimum inhibitory concentrations (MICs) of magnolol against phytopathogenic fungi.

Phytopathogenic fungus	MIC (μg/ml)	
	Magnolol	Cycloheximide
*Alternaria porri*	6.3	6.3
*Alternaria brassicicola*	6.3	6.3
*Botrytis cinerea*	25	50
*Colletotrichum cocodes*	6.3	1.6
*Fusarium oxysporum*	25	200
*Magnaporthe oryzae*	3.1	6.3
*Phytophthora infestans*	>100	1.6

**Fig 4:**
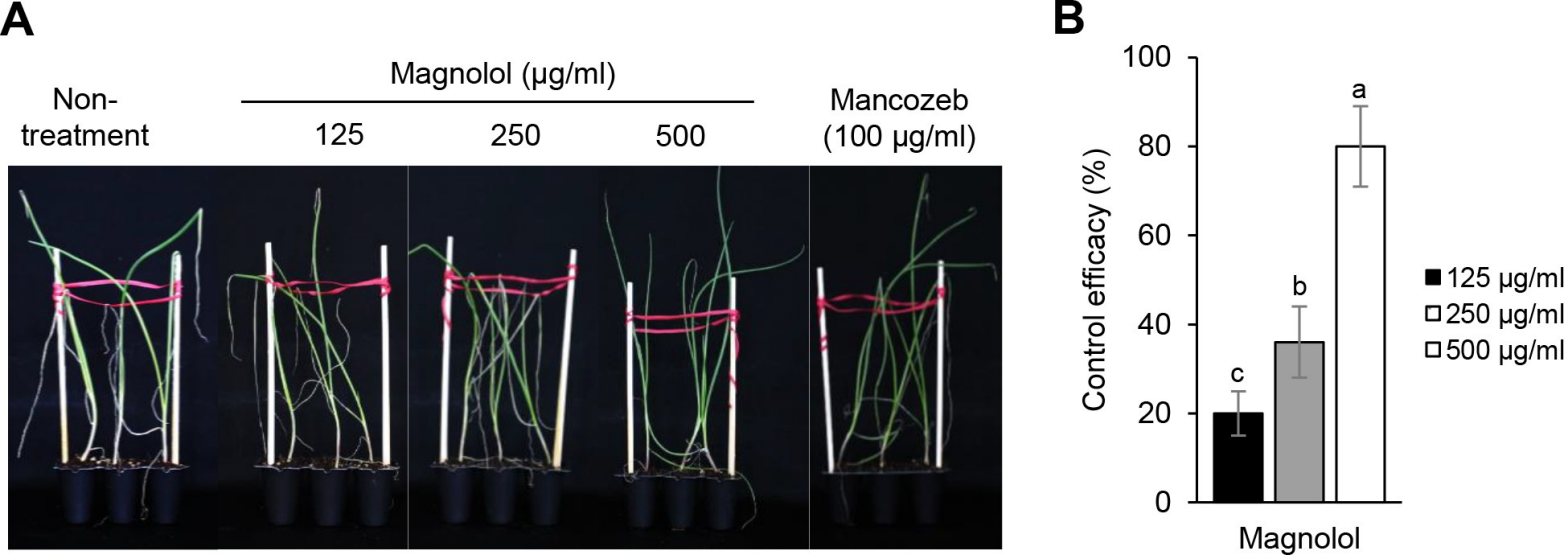
Effects of magnolol on the development of onion purple blotch disease. (A) Representatives of onion plants treated with magnolol. Treatments of mancozeb (100 μg/ml) and 1% methanol were used as positive and negative controls, respectively. Plants were inoculated with a spore suspension (1 × 10^5^ spores/ml) of *Alternaria porri* at 24 h after the magnolol treatment. (B) Control efficacy of magnolol against onion purple blotch disease. The bars represent the mean ± standard deviation of two runs with three replicates. Different letters indicate significant differences at *p* < 0.05.

To investigate the disease control efficacy of magnolol against onion purple blotch disease, it was applied to onion plants at a concentration of 125, 250, and 500 μg/ml. At a concentration of 500 μg/ml, the magnolol sufficiently controlled the development of onion purple blotch with a disease control value of 80% compared to the non-treatment control (Fig 4). At the concentration of 125 and 250 μg/ml, magnolol exhibited disease control values of less than 40%. In contrast, the fungicide mancozeb exhibited disease control values of 95% at a concentration of 100 μg/ml (Fig 4). There were no phytotoxic symptoms in this study by magnolol on the onion plants (S5 Fig).

### Inhibitory effects of magnolol on mitochondrial respiratory complexes

**Fig 5:**
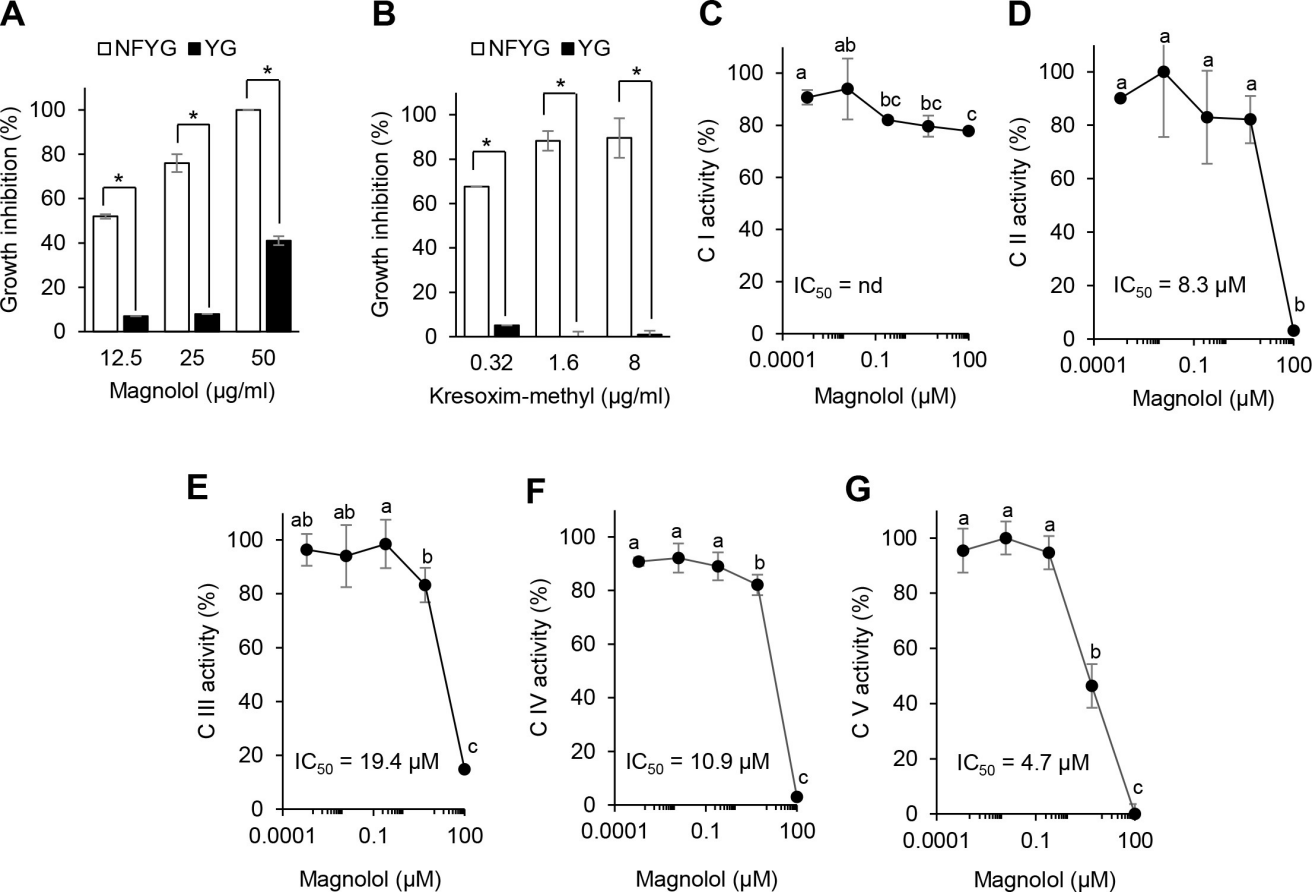
Effects of magnolol on mitochondrial respiratory. Growth comparison of *Saccharomyces cerevisiae* in NFYG and YG media containing magnolol (A) and kresoxim-methyl (B). (C–G) Effects of magnolol on the mitochondrial respiratory complexes I–V (C I–V). The values represent the mean ± standard deviation of two runs with three replicates. Different letters and asterisks (*) indicate significant differences at *p* < 0.05.

Considering that magnolol is more effective in spore germination than in mycelial growth (Fig 3), we investigated the respiratory inhibition of *S*. *cerevisiae* based on the growth inhibition in two different liquid media YG (containing a fermentable carbon source) and NFYG (containing a non-fermentable carbon source) containing magnolol. When magnolol was supplemented in a growth medium at a concentration of 12.5 and 25 μg/ml, the growth inhibition of *S*. *cerevisiae* grown in the NFYG medium by magnolol was approximately 8- and 9-fold higher than that of *S*. *cerevisiae* grown in the YG medium, respectively (Fig 5A). In contrast, when magnolol was supplemented in a growth medium at a concentration of 100 μg/ml, the growth inhibition of *S*. *cerevisiae* grown in the NFYG medium by magnolol was 2.5-fold higher than that of *S*. *cerevisiae* grown in the YG medium (Fig 5A). Although a high concentration (100 μg/ml) of magnolol did not show respiratory inhibition against *S*. *cerevisiae*, the results derived from the low concentrations of magnolol (12.5 and 25 μg/ml) were comparable to the inhibition results by a quinone outside inhibitor, the fungicide kresoxim-methyl (Fig 5A and 5B). Therefore, these results suggest that magnolol might possess an inhibitory activity against fungal respiration.

For the detailed inhibitory activity of magnolol on mitochondrial respiration, we measured the activity of each complex of the respiratory chain (OXPHOS complexes I to V) in the presence of magnolol (1–100 μM). As shown in Fig 5, magnolol inhibited complexes II, III, IV, and V with IC_50_ values of 8.3, 19.4, 10.9, and 4.7 μM, respectively. Although the activity of complex I was decreased by the magnolol treatment in a concentration-dependent manner, the IC_50_ values for complex I could not be determined (Fig 5C). Considering that the activity of complex I was 78% at the maximum test concentration of 100 μM, the results suggest that the maximum available concentration of magnolol for complex I was likely insufficient for the inhibitory activity to determine an IC_50_ value (Fig 5). In contrast, the complex specific inhibitors used as a positive control (rotenone for complex I, TTFA for complex II, antimycin A for complex III, KCN for complex IV, and oligomycin for complex V) suppressed each complex activity in a concentration-dependent manner (S6 Fig). Taken together, our results suggest that magnolol exhibits a broad range of inhibitory activity on the OXPHOS complexes, and in particular, complexes II–V might be more sensitive targets of magnolol than complex I.

## Discussion

Conventional chemical fungicides are effective for the control of various plant diseases, including *Alternaria* purple blotch. However, considering the unpredictable adverse effects of synthetic compounds in animals and humans, it is necessary to search for new safer and eco-friendly fungicides [28]. To discover natural antifungal resources on purple blotch of onion more efficiently, we established in this study the most favorable conditions for spore production of *A*. *porri* and the disease development. The results agreed with previous findings that a good production of spores can be provided on solid agar if the cultures receive physical stress and light stimulus [22, 23]. Our results can be useful for *in vitro* and *in vivo* antifungal assay to find antifungal compounds for the control of onion purple blotch caused by *A*. *porri*.

In this study, we found that the extract of *C*. *baviensis* leaves and stems was a natural antifungal resource against *A*. *porri* and identified the neolignan magnolol as a major antifungal substance from the extract. The plant species *C*. *baviensis* (Lec.) A.-Shaw is an endemic shrub of Vietnam, belonging to the flowering plant family Lauraceae [29]. From *Caryodaphnopsis* spp., it has been reported that several lignan compounds such as eupamatenoids and sesamin have been isolated from *C*. *baviensis* and *C*. *tonkinensis*, and the *C*. *baviensis* extract exhibited antibacterial activity against clinical *Bacillus* and *Staphylococcus* isolates with MICs of 64–128 μg/ml [30, 31]. However, there is still a lack of studies describing the *in vitro* and *in vivo* antifungal activities of *C*. *baviensis*. This study is the first report that the extract of *C*. *baviensis* exhibited a disease control efficacy for onion purple blotch, and magnolol was identified as a major antifungal substance from *C*. *baviensis*. During the isolation procedures of active compounds, we expected to isolate the isomers of magnolol such as honokiol and 4-*O*-methylhonokiol [26, 27, 30]. However, further activity-guided fractionation and spectroscopic analyses for finding other antifungal neolignans revealed that magnolol was the only major component in the antifungal fractions of the *C*. *baviensis* methanol extract.

Considering that magnolol is one of the well-known phytochemicals with a broad spectrum of antimicrobial activity, we investigated how other plant pathogenic fungi are sensitive to magnolol. As shown in Table 2, magnolol was highly active against *A*. *porri*, *A*. *brassiciciola*, *C*. *coccodes*, and *M*. *oryzae* with MICs of 3.1–6.3 μg/ml, which were compatible with those of antibiotic fungicide cycloheximide. These results suggest that magnolol may have a potential role as an antifungal agent to control plant disease with a broad spectrum. The previous study also supported the effectiveness of magnolol in controlling plant diseases [32]. Choi et al. (2009) [32] showed that magnolol isolated from a *Magnolia obovata* extract has a broad-spectrum antifungal activity against phytopathogenic fungi and an effective disease control efficacy for rice blast, tomato late blight, wheat leaf rust, and pepper anthracnose. In terms of safety issues, there have been no reports on side effects from the ingestion of herbal formulations containing magnolol [33]. Previous mutagenic and genotoxic studies have also shown that magnolol does not give rise to any concerns for human health [34]. When taken orally, >90% of magnolol is rapidly excreted in the feces and urine, and only 1% of the oral dose remains circulating in the free form for 12–16 h without any hepatic side effects [35]. Furthermore, magnolol has been regarded as a safe material for humans by different food safety authorities [33]. Based on the safety issue and disease control efficacy found in this study, *C*. *baviensis* extract containing magnolol could be considered as one of the most effective and eco-friendly resources to control onion purple blotch. To develop biopesticide using *C*. *baviensis* extract, research such as formulation, field evaluations, and large-scale production requires further elucidation. Additionally, the investigation through which *C*. *baviensis* extract can be used as stand-alone products or in combination with current synthetic fungicides might strengthen integrated disease management programs.

Magnolol is a representative neolignan compound isolated from the cortex of *Magnolia* plants, which has been used for a long time in traditional oriental medicine [36–38]. It has been reported that magnolol exhibits promising antimicrobial activities against phytopathogenic fungi and bacteria [32, 37]. The antifungal and antimycotoxigenic activities of magnolol against *Alternaria* spp. have also been highlighted [26, 36, 39]. When *A*. *alternata* was grown on medium containing magnolol, the mycelial growth of *A*. *alternata* was inhibited, and the hyphae became shrunken and wrinkled because of changes in cell permeability by the impairment of plasma membranes [36, 40]. In addition, it has been reported that magnolol causes a significant decrease in the production of *Alternaria* mycotoxins (e.g., alternariol, alternariol monomethyl ether, and tenuazonic acid) and in the transcription level of genes involved in their biosynthesis [39, 40]. In gray mold *B*. *cinerea*, Cui et al. (2021) [41] showed that magnolol induces mitochondrial damage and excessive ROS generation, followed by impaired membrane integrity and attenuated cell vitality, resulting in reduced mycelial growth and virulence. However, relatively little is known mechanistically about how magnolol inhibits respiratory chain complexes and affects mitochondrial ROS generation. Therefore, we investigated the direct inhibitory effect of magnolol against individual OXPHOS complex and clarified the potent and broad inhibitory action of magnolol on complexes I–V.

Honokiol, an isomer of magnolol, also has been reported to have an antifungal property by inducing mitochondrial dysfunction and ROS generation in fungal pathogens such as *A*. *alternata*, *B*. *cinerea*, and *Candida albicans* [36, 41, 42]. Sun et al. (2017) [42] suggested that the OXPHOS complex I is a potent target of honokiol which induces ROS production and apoptosis in both fungal and human cancer cells. Similarly, our results also showed that the complex I activity was decreased by magnolol in a concentration-dependent manner. However, rather than complex I, we observed that complexes II–V are more sensitive to magnolol. Although the difference in chemical structure between magnolol and honokiol may not influence their biological activities, the different activities for the OXPHOS complexes require further study, particularly with *A*. *porri*.

In summary, our study presented the most favorable culture conditions for spore production of *A*. *porri* and the disease development condition for purple blotch for efficient *in vitro* and *in vivo* antifungal assays against *A*. *porri*. Of the tested plant extracts, we also found that the *C*. *baviensis* methanol extract showed a promising *in vitro* antifungal activity against *A*. *porri*. As a major antifungal component of the extract, the neolignan magnolol was identified for the first time from *C*. *baviensis*. The spore germination and mycelial growth of *A*. *porri* and the development of onion purple blotch were significantly reduced by the magnolol treatment. Furthermore, we found that the modes of inhibitory action of magnolol were associated with mitochondrial dysfunction in the electron transport chain complexes. Taken all together, these observations provide new insights into the potential of *C*. *baviensis* and magnolol as an active ingredient for the control of *Alternaria* pathogens in crops and agro-products.

## Supporting information

S1 Fig Culture plates by mechanical treatments. Colony surface was scraped by the blade (M1) and brush (M2), or cut into grid patterns with 2-mm and 5-mm spacing by a scalpel blade (M3 and M4).
(PDF)

S2 Fig Symptoms of onion purple blotch. Plants were inoculated with a spore suspension (1 × 10^5^ spores/ml) of *Alternaria porri*, and photos were taken 5 dpi. White arrows indicate symptoms by *A*. *porri*.
(PDF)

S3 Fig Isolation scheme of active compounds from the methanol extract of *Caryodaphnopsis baviensis*. (PDF)

S4 Fig HMBC correlations for compound CB1. (PDF)

S5 Fig Onion plants treated with magnolol. Photos were taken at 2 days after treatment of magnolol (500 μg/ml). There were no phytotoxic symptoms by magnolol.
(PDF)

S6 Fig Effect of complex specific inhibitors on the individual mitochondrial respiratory complexes (CI–CV). Rotenone, thenoyltrifluoroacetone (TTFA), antimycin A, potassium cyanide (KCN), and oligomycin were used as positive controls. The experiment was conducted twice with three replicates.
(PDF)

S1 Table ^1^H- and ^13^C-NMR spectra of compound CB1. (PDF)
