# Supplementary material for: Characterization of Alternaria porri causing onion purple blotch and its antifungal compound magnolol identified from Caryodaphnopsis baviensis
Source: PLoS One. 2022 Jan 20;17(1):e0262836. doi: 10.1371/journal.pone.0262836 (PMC8775252; doi:10.1371/journal.pone.0262836)
Supplement: S1 Fig — Colony surface was scraped by the blade (M1) and brush (M2), or cut into grid patterns with 2-mm and 5-mm spacing by a scalpel blade (M3 and M4). (PDF) [file pone.0262836.s001.pdf]

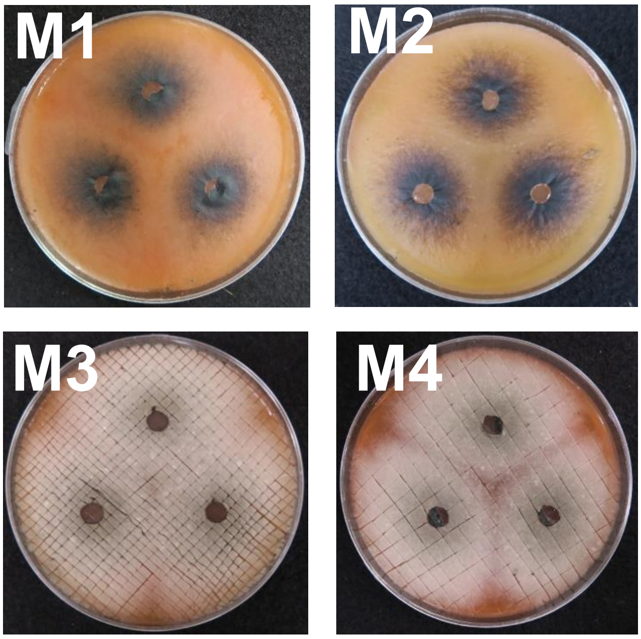

S1 Fig. Culture plates by mechanical treatments. Colony surface was scraped by the blade (M1) and brush (M2), or cut into grid patterns with 2-mm and 5-mm spacing by a scalpel blade (M3 and M4).
