# Supplementary material for: Characterization of Alternaria porri causing onion purple blotch and its antifungal compound magnolol identified from Caryodaphnopsis baviensis
Source: PLoS One. 2022 Jan 20;17(1):e0262836. doi: 10.1371/journal.pone.0262836 (PMC8775252; doi:10.1371/journal.pone.0262836)
Supplement: S2 Fig — Plants were inoculated with a spore suspension (1 × 10^5^ spores/ml) of Alternaria porri, and photos were taken 5 dpi. White arrows indicate symptoms by A. porri. (PDF) [file pone.0262836.s002.pdf]

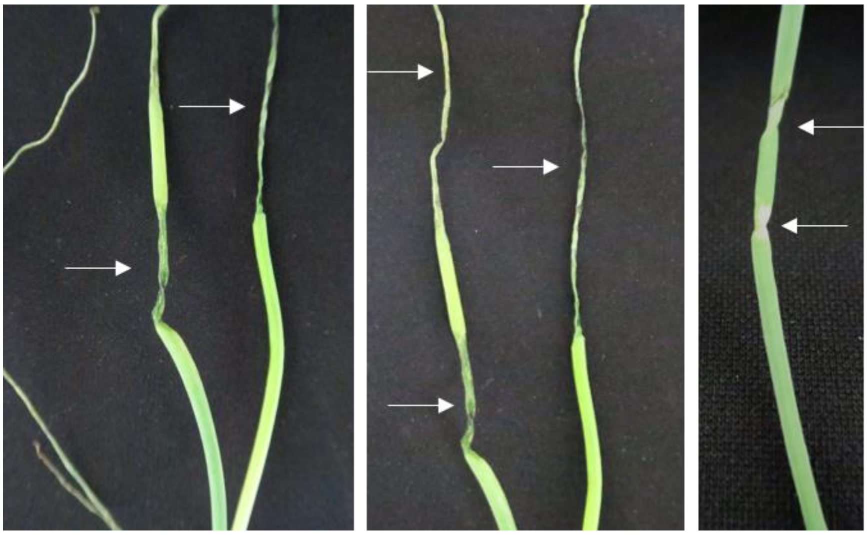

**S2 Fig. Symptoms of onion purple blotch.** Plants were inoculated with a spore suspension ($1 \times 10^5$ spores/ml) of *Alternaria porri*, and photos were taken 5 dpi. White arrows indicate symptoms by *A. porri*.
