# Supplementary figures and images for: Characterization of Alternaria porri causing onion purple blotch and its antifungal compound magnolol identified from Caryodaphnopsis baviensis
Source: PLoS One. 2022 Jan 20;17(1):e0262836. doi: 10.1371/journal.pone.0262836 (PMC8775252; doi:10.1371/journal.pone.0262836)

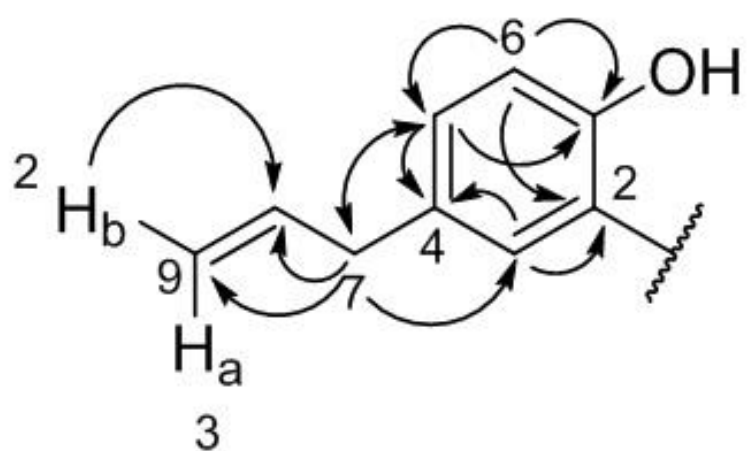

S4 Fig. HMBC correlations for compound CB1.

Supplement: S4 Fig — (PDF) [file pone.0262836.s004.pdf]
