# Supplementary material for: Characterization of Alternaria porri causing onion purple blotch and its antifungal compound magnolol identified from Caryodaphnopsis baviensis
Source: PLoS One. 2022 Jan 20;17(1):e0262836. doi: 10.1371/journal.pone.0262836 (PMC8775252; doi:10.1371/journal.pone.0262836)
Supplement: S5 Fig — Photos were taken at 2 days after treatment of magnolol (500 μg/ml). There were no phytotoxic symptoms by magnolol. (PDF) [file pone.0262836.s005.pdf]

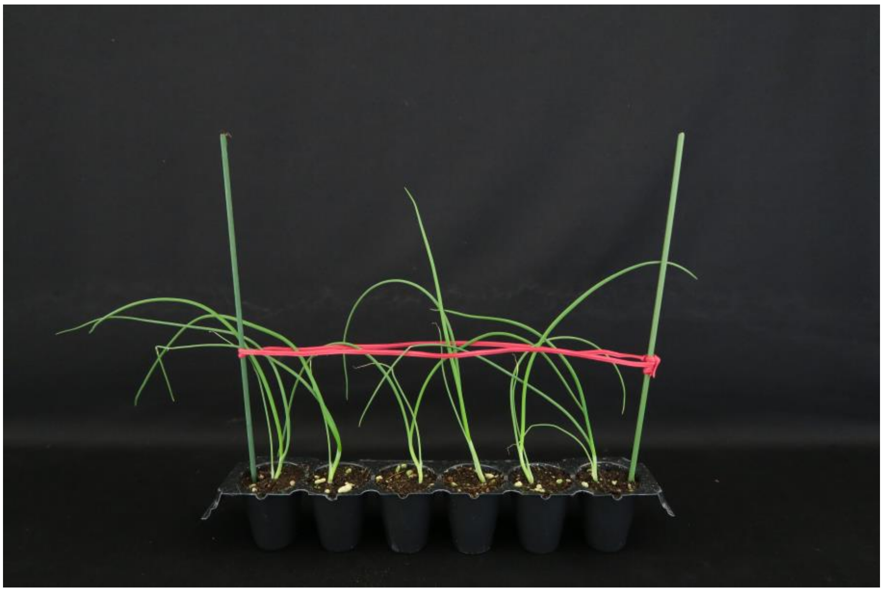

S5 Fig. Onion plants treated with magnolol. Photos were taken at 2 days after treatment of magnolol (500 μg/ml). There were no phytotoxic symptoms by magnolol.
