# Supplementary material for: Characterization of Alternaria porri causing onion purple blotch and its antifungal compound magnolol identified from Caryodaphnopsis baviensis
Source: PLoS One. 2022 Jan 20;17(1):e0262836. doi: 10.1371/journal.pone.0262836 (PMC8775252; doi:10.1371/journal.pone.0262836)
Supplement: S6 Fig — Rotenone, thenoyltrifluoroacetone (TTFA), antimycin A, potassium cyanide (KCN), and oligomycin were used as positive controls. The experiment was conducted twice with three replicates. (PDF) [file pone.0262836.s006.pdf]

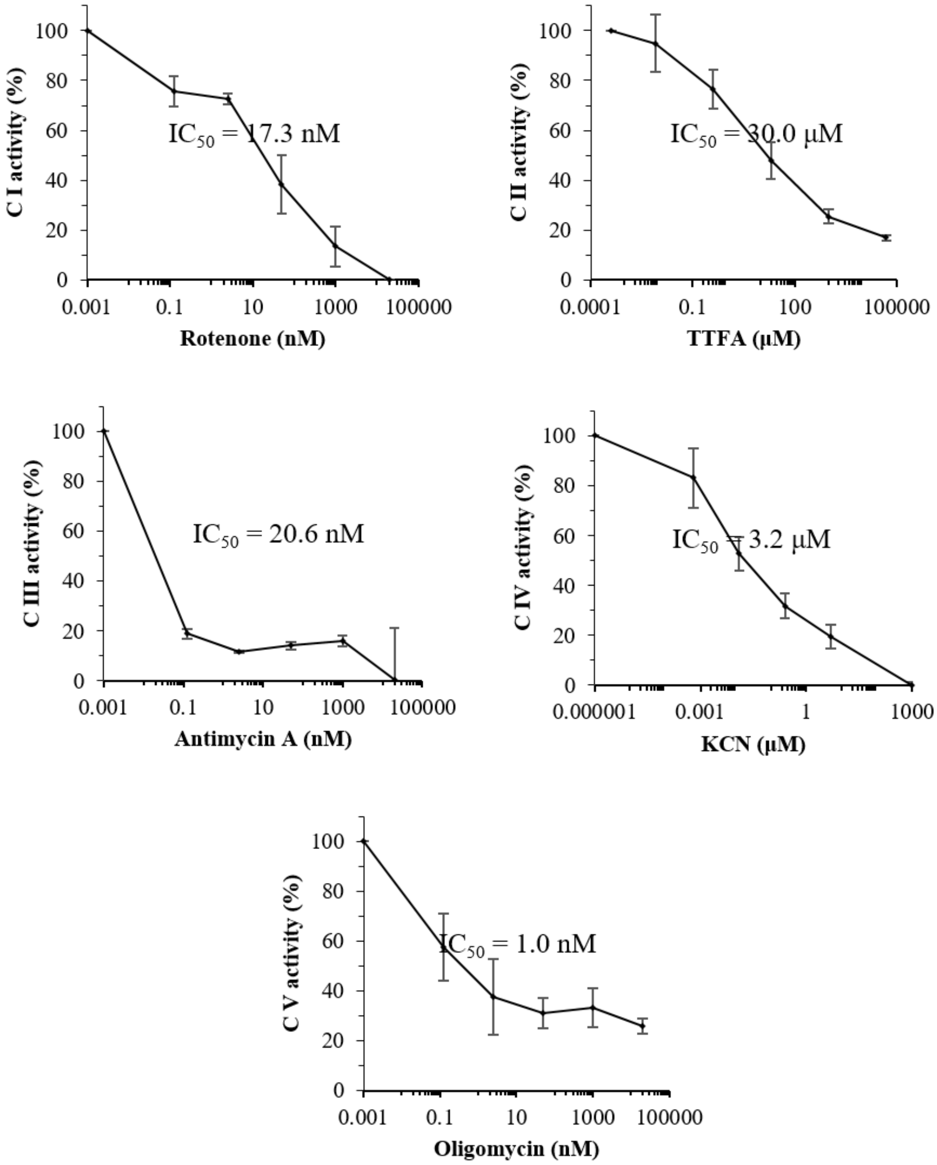

S6 Fig. Effect of complex specific inhibitors on the individual mitochondrial respiratory complexes (CI–CV). Rotenone, thenoyltrifluoroacetone (TTFA), antimycin A, potassium cyanide (KCN), and oligomycin were used as positive controls. The experiment was conducted twice with three replicates.
