# Supplementary material for: Characterization of Alternaria porri causing onion purple blotch and its antifungal compound magnolol identified from Caryodaphnopsis baviensis
Source: PLoS One. 2022 Jan 20;17(1):e0262836. doi: 10.1371/journal.pone.0262836 (PMC8775252; doi:10.1371/journal.pone.0262836)
Supplement: S1 Table — (PDF) [file pone.0262836.s007.pdf]

S1 Table. ^{1}H- and ^{13}C-NMR spectra of compound CB1*

Position	δ_C, Type	δ_H, mult. (*J*)
1, 1’	153.2, C	
2, 2’	127.6, C	
3, 3’	133.2, CH	7.03, dt (2.4, 0.5)
4, 4’	132.7, C	
5, 5’	129.8, CH	7.02, ddt (8.1, 2.3, 0.6)
6, 6’	117.4, CH	6.85, d (8.1)
7, 7’	40.4, CH_2	3.32, d (6.9, 1.6)
8, 8’	139.4, CH	5.96, ddt (16.8, 10.1, 6.7)
9, 9’	115.6, CH_2	5.05, ddt (17.0, 2.2, 1.6); 5.01, ddt (10.1, 2.1, 1.3)

*Recorded in CD_3OD at 500 MHz; δ in ppm and *J* in Hz
